# Impact of a Glaucoma Severity Index on Results of Trabectome Surgery: Larger Pressure Reduction in More Severe Glaucoma

**DOI:** 10.1371/journal.pone.0151926

**Published:** 2016-03-23

**Authors:** Ralitsa T. Loewen, Pritha Roy, Hardik A. Parikh, Yalong Dang, Joel S. Schuman, Nils A. Loewen

**Affiliations:** Department of Ophthalmology, School of Medicine, University of Pittsburgh, Pittsburgh, Pennsylvania, United States of America; Casey Eye Institute, UNITED STATES

## Abstract

**Purpose:**

To stratify outcomes of trabectome-mediated ab interno trabeculectomy (AIT) by glaucoma severity using a simple and clinically useful glaucoma index. Based on prior data of trabectome after failed trabeculectomy, we hypothesized that more severe glaucoma might have a relatively more reduced facility compared to mild glaucoma and respond with a larger IOP reduction to trabecular meshwork ablation.

**Methods:**

Patients with primary open angle glaucoma who had undergone AIT without any other same session surgery and without any second eye surgery during the following 12 months were analyzed. Eyes of patients that had less than 12 months follow up or were diagnosed with neovascular glaucoma were excluded. A glaucoma index (GI) was created to capture glaucoma severity based on visual field, number of preoperative medications, and preoperative IOP. Visual field (VF) was separated into 3 categories: mild, moderate, and advanced (assigned 1, 2, and 3 points, respectively). Preoperative number of medications (meds) was divided into 4 categories: ≤1, 2, 3 or ≥4, and assigned with a value of 1 to 4. Baseline IOP (IOP) was divided into 3 categories: <20 mmHg, 20–29 mmHg, and greater than 30 mmHg and assigned with 1 to 3 points. GI was defined as IOP × meds × VF and separated into 4 groups: <6 (Group 1), 6–12 (Group 2), >12–18 (Group 3) and >18 (Group 4). Linear regression was used to determine if there was an association between GI group and IOP reduction after one year or age, gender, race, diagnosis, cup to disc (C/D) ratio, and Shaffer grade.

**Results:**

Out of 1340 patients, 843 were included in the analysis. The GI group distribution was GI1 = 164, GI2 = 202, GI3 = 260, and GI4 = 216. Mean IOP reduction after one year was 4.0±5.4, 6.4±5.8, 9.0±7.6, 12.0±8.0 mmHg for GI groups 1 to 4, respectively. Linear regression showed that IOP reduction was associated with GI group after adjusting for age, gender, race, diagnosis, cup to disc ratio, and Shaffer grade. Each GI group increase of 1 was associated with incremental IOP reductions of 2.95±0.29 mmHg. Success rate at 12 months was 90%, 77%, 77%, and 71% for GI groups 1 to 4. The log-rank test suggested significant differences between GI groups.

**Conclusion:**

A simple glaucoma index, GI, was created to capture glaucoma severity and a relative resistance to treatment. A higher GI was associated with a larger IOP reduction in trabectome surgery. This indicates that there is a role for AIT beyond mild glaucoma and ocular hypertension.

## Introduction

The trabecular meshwork (TM) is the primary site of outflow resistance in open angle glaucoma [1]. Plasma-mediated ablation of this tissue in ab interno trabeculectomy (AIT) using the trabectome increases outflow facility in different types of glaucomas [2–4]. We hypothesized that an eye with a more diseased trabecular meshwork has a higher outflow resistance and might therefore experience a larger intraocular pressure (IOP) reduction than one with only a mild outflow resistance. The preoperative number of medications and visual field status might similarly reflect the severity of trabecular meshwork disease and its resistance in open angle glaucoma. A previous study suggested that patients with a failed trabeculectomy and advanced visual field damage had a larger IOP decrease from trabectome surgery than patients with a less advanced visual field loss but these differences were not statistically significant due to sample size [5].

In the present study we created a simple glaucoma index that combines preoperative IOP, number of preoperative medications and visual field damage to capture relative glaucoma severity and resistance to treatment. This index was used to compare outcomes of AIT stratified by glaucoma index.

## Methods

Data for this study were analyzed with approval by the Institutional Review Board of the University of Pittsburgh, in accordance with the Declaration of Helsinki and the Health Insurance Portability and Accountability Act. No informed consent was necessary for this retrospective, observational cohort study. Patient records were anonymized and de-identified prior to analysis. The specific target IOP was set on a case-by-case basis by the individual treating physician and the maximum IOP was estimated to prevent further nerve damage. Patients who were followed for less than 12 months or diagnosed with neovascular glaucoma were excluded. Indications for AIT consisted of worsening glaucoma on maximally tolerated topical therapy. Anterior chamber angles in all patients were graded by Shaffer grade (SG) [3,6], a classification system in which ‘0’ to ‘slit’ represents a totally or partially closed angle with potential for angle closure that is present or very likely, ‘1’ an angle width of 10° (very narrow) and closure potential that is probable, ‘2’ representing 20° and possible potential for closure, ‘3’ standing for 20° to 45° with unlikely closure and grade ‘4’ indicating a wide open angle and improbable potential for angle closure.

The glaucoma index, GI, was created as a variable based on visual field, number of preoperative medications, and preoperative IOP. Visual field was separated into 4 categories: up to mild, up to moderate, up to advanced, and more than advanced visual field damage [7], which were assigned 1, 2, 3 and 4 points, respectively. Preoperative number of medications were divided into 4 categories: 0–1, 2, 3 or 4+, and assigned with a value of 1 to 4, respectively. Baseline IOP was divided into 3 categories: <20 mmHg, 20–29 mmHg, 30–39 mmHg, and greater than 40 mmHg and assigned with 1 to 4 points, respectively. These categories were chosen based on IOP distribution and designed not to underrate low pressure glaucoma. GI was then defined as preoperative IOP × preoperative number of medications × VF. GI was separated into 4 groups: <6 (Group 1), 6–12 (Group 2), >12–18 (Group 3) and >18 (Group 4). A power calculation indicated that 82 patients would allow to detect a GI group IOP difference or 3 mmHg for a medium-sized effect of 0.3 with a power of 80%. Linear regression was used to determine if there was an association between GI group and IOP reduction after one year. An identical analysis was performed only for patients with primary open angle glaucoma.

Baseline characteristics were compared by the Kruskal-Wallis and chi square tests for continuous and categorical variables between GI groups, respectively. Univariate linear regression was performed first and those variables that were found to be statistically significant were included into multivariate regression. The term “success rate” was used here instead of the statistics term “survival” and defined as IOP≤21 mmHg, at least 20% IOP reduction from baseline in any two consecutive visits after three months, and no secondary glaucoma or lens surgery. Log-rank test was used to compare success distributions of GI groups. Continuous data was reported as mean ± standard deviation.

## Results

**Fig 1:**
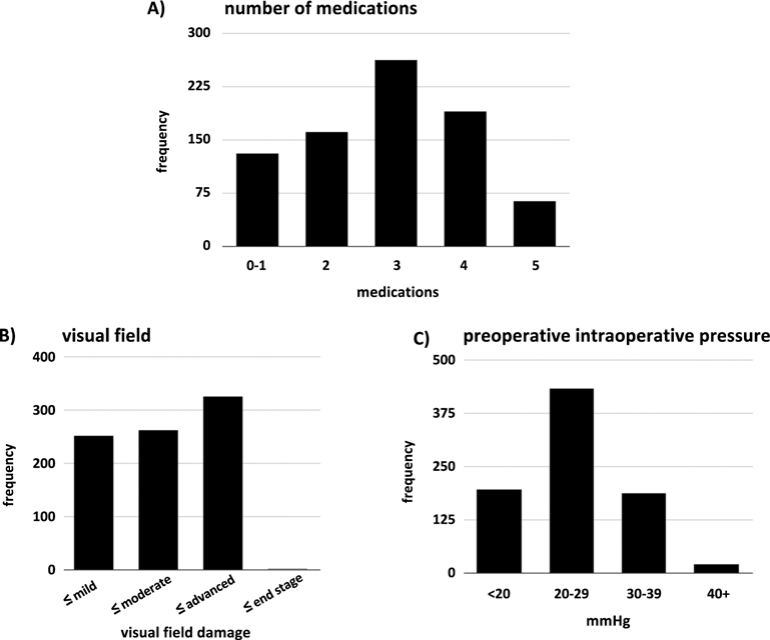
Distribution of Glaucoma Index Variables. A) Visual field score distribution included a relatively large number of eyes with advanced visual field damage. B) Three medications was the most common median number of eye drops used. C) Most patients had a preoperative intraocular pressure in the range of 20 to 29 mmHg.

A total of 842 eyes were analyzed. The average age of all patients was 66.4±15.8 years. Variables used to compute GI were 1) preoperative IOP, 2) preoperative number of medications and 3) visual field status. The average preoperative IOP for all patients was 25.1±6.7 mmHg, the average medication count was 2.9±1.0 and the most common visual field was advanced. The baseline IOP categories were chosen as described above because of the distribution (Fig 1).

Only 22 eyes had an IOP above 40 mmHg and had to be excluded because of an insufficient power to contribute to a separate group. There was no significant age difference among GI groups **(Table 1)**.

**Table 1:** Demographics and Biometrical Data.

	GI Group 1 (n = 164)	GI Group 2(n = 202)	GI Group 3(n = 260)	GI Group 4(n = 216)	p-value
Age					0.26
Mean±SD	69±12	66±14	67±17	64±19	
Range	36–92	18–88	18–96	18–96	
Gender					<0.01
Female	102 (62%)	117 (58%)	123 (47%)	99 (46%)	
Male	59 (36%)	84 (42%)	136 (52%)	109 (50%)	
NR	3 (2%)	1 (0%)	1 (0%)	8 (4%)	
Ethnicity					<0.01
African American	15 (9%)	10 (5%)	15 (6%)	11 (5%)	
Asian	26 (16%)	47 (23%)	74 (28%)	76 (35%)	
Caucasian	93 (57%)	114 (56%)	146 (56%)	97 (45%)	
Hispanic	22 (13%)	15 (7%)	14 (5%)	19 (9%)	
Other	8 (5%)	16 (8%)	11 (4%)	13 (6%)	
Diagnosis					<0.01
POAG	136 (83%)	154 (76%)	177 (68%)	136 (63%)	
Pseudoexfoliation	10 (6%)	20 (10%)	39 (15%)	29 (13%)	
Pigment Dispersion	9 (5%)	7 (3%)	8 (3%)	5 (2%)	
Steroid Induced	3 (2%)	12 (6%)	24 (9%)	34 (16%)	
Others	6 (4%)	9 (4%)	12 (5%)	12 (6%)	
VA (logMar)					<0.01
Mean±SD	0.23±0.34	0.24±0.36	0.32±0.53	0.44±0.58	
Range	-0.18–2.12	-0.19–2.00	-0.19–3.00	-0.19–3.00	
Disc C/D					<0.01
Mean±SD	0.69±0.16	0.75±0.15	0.73±0.18	0.83±0.11	
Range	0.1–0.95	0.3–1.0	0.1–1.0	0.3–1.0	
Lens Status					0.05
Phakic	95 (58%)	115 (57%)	132 (51%)	96 (44%)	
Pseudophakic	54 (33%)	77 (38%)	114 (44%)	103 (48%)	
Aphakic	1 (1%)	1 (0%)	0 (0%)	3 (1%)	
NR	14 (9%)	9 (4%)	14 (5%)	14 (6%)	
Shaffer Grade					0.16
I	1 (1%)	6 (3%)	3 (1%)	1 (0%)	
II	9 (5%)	10 (5%)	14 (5%)	16 (7%)	
III	42 (26%)	58 (29%)	80 (31%)	69 (32%)	
IV	99 (60%)	97 (48%)	131 (50%)	103 (48%)	
NR	13 (8%)	31 (15%)	32 (12%)	27 (12%)	

GI1: GI<6, GI2: 6≤GI<12, GI3: 12≤GI<18, GI4: GI≥18

GI1 had a significantly lower portion of male patients while the other groups were more gender balanced. Most patients were Caucasians followed by Asians and then by Hispanics and African Americans at comparable percentages. Seventy-one percent had the diagnosis of primary open angle glaucoma which was more common in GI1. Steroid induced glaucoma increased in frequency from GI1 to GI4 eightfold. Visual acuity decreased from GI1 to GI4 to approximately half of that of GI1. The cup to disc (C/D) ratio increased significantly by glaucoma severity index from 0.69 to 0.83. There were more pseudophakic eyes in GI4 compared to the other groups and most of the phakic eyes were found in GI1 (p = 0.05). There were no significant differences in distribution of degree of angle opening among GI groups and approximately 80% of eyes had a Shaffer grade above 2 indicating a wide open angle.

**Fig 2:**
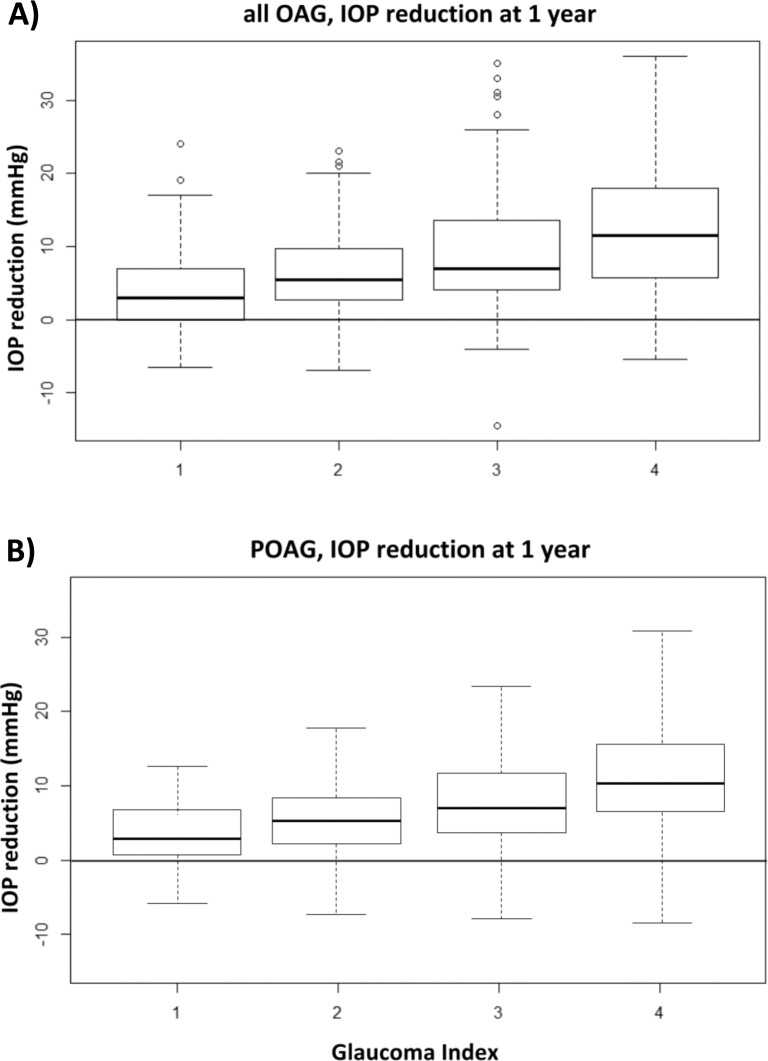
IOP reduction at one year by glaucoma index. Patients with a higher glaucoma index (GI) had a larger absolute reduction of intraocular pressure (IOP).

Mean IOP reduction at one year was 4.0±5.4, 6.4±5.8, 9.0±7.6, 12.0±8.0 mmHg for GI groups 1 to 4, respectively (Fig 2), with significant intergroup differences (p<0.05). There was a larger range of IOP reduction in higher groups.

**Fig 3:**
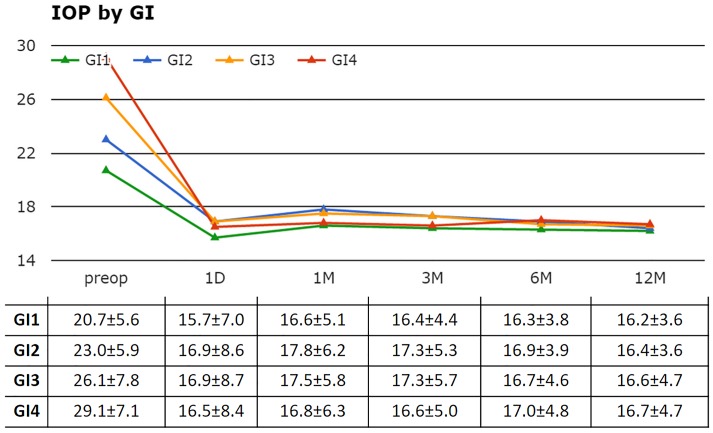
Individual IOPs by glaucoma index (GI). A higher GI was associated with a higher preoperative IOP while postoperative IOPs were similar (table: mean±SD).

A higher preoperative IOP was associated with a higher GI group assignment but the postoperative IOPs were not significantly different at any visit and often indistinguishable (Fig 3).

**Fig 4:**
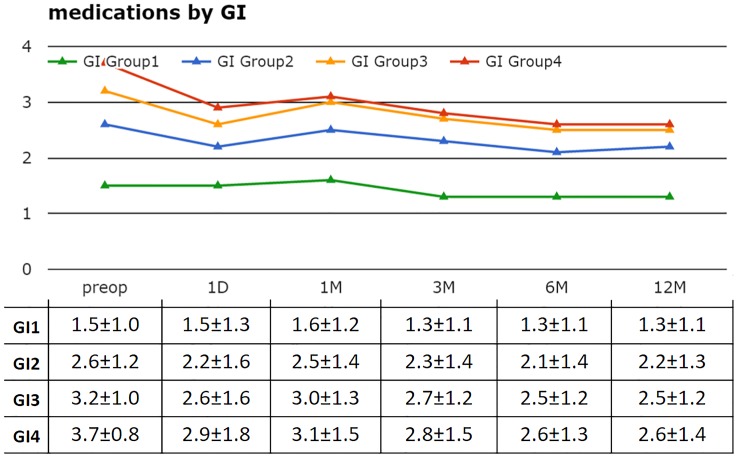
Mean number of medications by GI. While there was an overall decrease in the number of medications in all groups, GI3 and GI4 showed the most significant changes (p<0.05) after 6 months postoperatively.

Similarly, the higher the GI group assignment, the higher the preoperative medication count (Fig 4). The higher GI groups, GI4 and GI3, had a significantly lower average medication count at 6 and 12 months.

**Fig 5:**
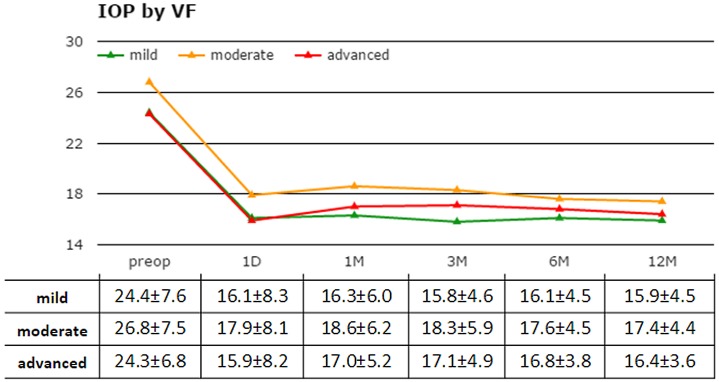
Intraocular pressure by visual field. While patients with moderate visual field loss had slightly higher IOPs prior to surgery, they were not different after day 1.

**Fig 6:**
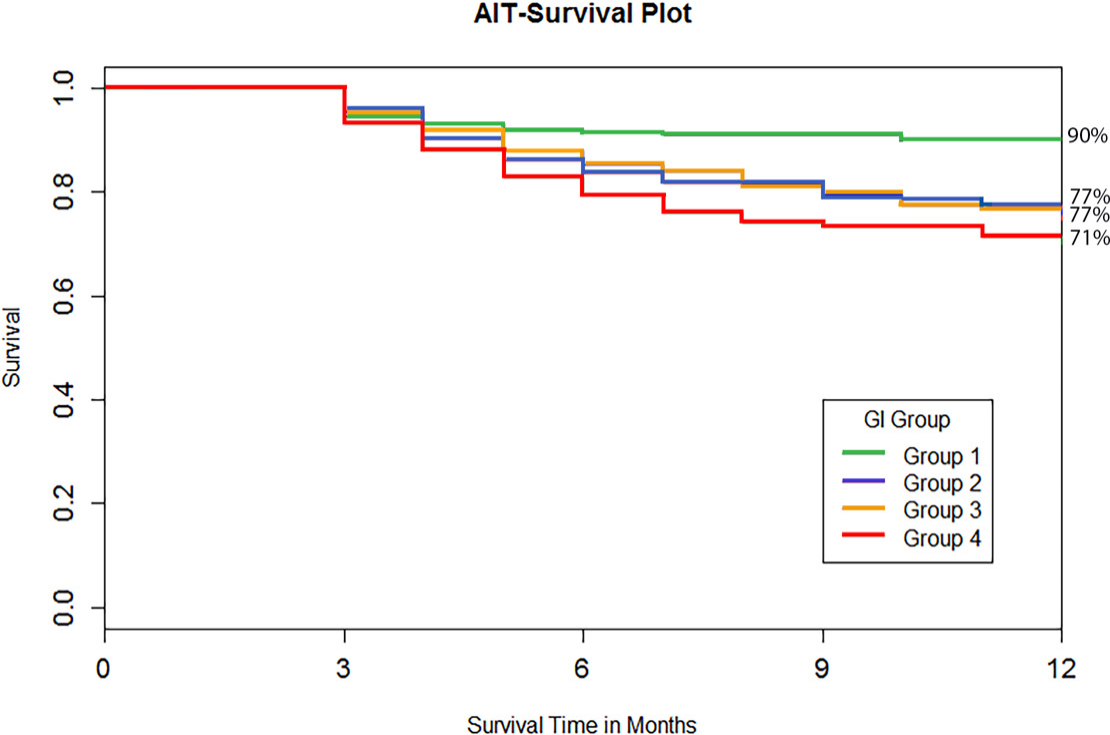
Success plot by glaucoma index. Subjects in GI1 had the best success while GI2 through GI4 were not significantly different.

Patients with moderate visual field damage had a slightly higher average preoperative and day 1 IOP compared to patients with mild or advanced visual field loss (Fig 5). Differences were not significant thereafter (p>0.05). Compared to GI2 through GI4, GI1 had a better success rate of 90%, the highest of all groups (Fig 6). This was followed by GI2 and GI3 with 77% followed closely by GI4 with 71%.

Univariate linear regression (Table 2), which was performed first, identified age, male gender, Hispanic ethnicity, diagnosis of pseudoexfoliation and steroid induced glaucoma, and C/D ratio to be most significant. These were included in the multivariate regression.

**Table 2:** Univariate Regression.

	Coefficient	Standard Error	p-value
Age	-0.07	0.02	<0.01
Male	1.55	0.58	<0.01
Ethnicity			
Asian	0.89	1.29	0.49
Caucasian	1.25	1.28	0.33
Hispanic	4.44	1.62	<0.01
Other	0.37	1.66	0.82
Diagnosis			
Other	2.45	1.44	0.09
Pseudoexfoliation Glaucoma	2.75	0.94	<0.01
Pigmentary Dispersion	1.61	1.82	0.37
Steroid Induced	5.77	1.17	<0.01
C/D Ratio	-5.94	2.07	<0.01
Shaffer Grade	-1.87	1.16	0.17
Lens			
Aphakic	-2.42	3.81	0.53
Pseudophakic	-1.42	0.76	0.08

In the multivariate regression analysis (Table 3), GI group, age, Hispanic ethnicity, steroid induced glaucoma, and C/D ratio were found to be significantly associated with IOP reduction. For patients that differ in GI groups by 1, patients in the higher GI group are expected to have a further incremental IOP reduction of 2.95±0.29 mmHg than patients in the next lower GI group. Success rate at 12 months was 90%, 77%, 77%, and 71% for GI groups 1 to 4. Log-rank test indicated a statistically significant success rate difference between GI groups. Patients in lower GI groups had a higher success rate than those in higher GI groups. The regression analysis had a power of 0.95.

**Table 3:** Multivariate Regression for All Open Angle Glaucomas.

	Coefficient	Standard Error	p-value
GI group	3.02	0.30	<0.01
Age	-0.05	0.02	0.01
Male	0.52	0.55	0.35
Ethnicity			
Asian	-1.20	1.17	0.31
Caucasian	1.21	1.19	0.31
Hispanic	4.33	1.49	< 0.01
Other	0.06	1.46	0.97
Diagnosis			
Other	1.84	1.37	0.18
Pigmentary Dispersion	0.40	1.73	0.82
Pseudoexfoliative Glaucoma	1.78	0.92	0.06
Steroid Induced	3.37	1.32	0.02
C/D Ratio	-9.79	2.06	<0.01

The analogous analysis of the above of only primary open angle glaucoma patients indicated a similar greater IOP reduction of IOP with higher GI group (Fig 3B). We also determined the relative IOP reduction as a function of preoperative IOP (S1 Fig). In analogy to the absolute reduction, the percentage decrease was the larger the higher the group index.

The results of the multivariate analysis for POAG only (Table 4) also found GI group to be associated with IOP reduction in a similar fashion as all patients (Table 3). After adjusting for gender, race and cup-to-disc ratio, cases in one GI group higher had an extra 2.98±0.28 mmHg reduction in IOP than those in one GI group lower. Males experienced an additional 1.32 mmHg IOP reduction compared to females with the same GI group, race and C/D. Hispanics had a greater IOP reduction of 4.18 mmHg when compared to African Americans after adjusting for all other variables. Patients with a higher C/D experienced a lesser IOP decrease than those with lower C/D after adjusting for GI group, gender and race.

**Table 4:** Multivariate Regression for Primary Open Angle Glaucoma.

	Coefficients	Standard Error	p-value
GI group	2.98	0.26	<0.01
Male	1.32	0.60	0.03
Race			
Asian	-0.88	1.12	0.43
Caucasian	0.64	1.06	0.54
Hispanic	4.18	1.45	<0.01
Other	-1.20	1.62	0.46
C/D	-11.74	1.99	<0.01

## Discussion

Despite recent efforts [8] to incorporate glaucoma severity into clinical practice to include treatment guidelines and outcome measures [9], most staging systems only consider visual field function [7], optic nerve damage [10] or a combination of visual field and retinal nerve fiber layer loss [11]. Richardson described an early system to combine treatment goals and modalities as well as functional damage into a glaucoma stage [12]. Because few topical glaucoma medications existed at that time, the number of topical medications was not a useful measure to gauge resistance to treatment and relate this to surgical success. Reduction of medication is now commonly used as a primary outcomes measure that is reported together with IOP, both in traditional glaucoma surgery [13] and in micro-incisional glaucoma surgeries [3,14]. A stratification of glaucoma surgical outcomes by glaucoma severity has not been formally examined and published. This study fills an important knowledge gap in by providing this data for a form of modern glaucoma surgery that is minimally invasive and has a considerably better safety profile [4].

The goal of this study was to examine whether clinical open angle glaucoma severity, as captured by a simple glaucoma index, is correlated to IOP reduction. Glaucoma with a high IOP despite several anti-glaucoma medications and worsening visual field may indicate a high treatment resistance or, more specifically, a highly reduced outflow facility. We hypothesized that eyes with a higher glaucoma index would show a larger IOP reduction following TM ablation compared to eyes with less severe outflow resistance. This is indeed the case and consistent with the consensus that the TM is the site of the highest outflow resistance in most open angle glaucomas. While postoperative IOPs were highly similar in all GI groups, the most severe groups also experienced the largest reduction in medications. Such behavior is predicted by the Goldmann equation (IOP = F/C + Pv—U (aqueous humor formation/facility + episcleral venous pressure—uveoscleral outflow)) because a large facility “C” (TM is absent) will result in a very small “F/C” so that IOP is only limited by the episcleral venous pressure and uveoscleral outflow. Consistent with this, two studies have reported a surprisingly high success rate of trabectome surgery after failed tube shunts [15] and after failed trabeculectomy [5].

As could be expected, GI1, indicative of mild glaucoma, had the best success of all groups. Interestingly, the remaining GI2 and 3 had an almost identical success course and GI4 was only slightly worse. The success rate observed here is very similar to that of other studies [16,17] or better [18]. This suggests that greater glaucoma severity may be associated with an increase in surgical failure rates.

The data presented here matches established glaucoma risk factors and provides some validation: GI1 had disproportionately more female patients. This may reflect the preponderance of women among patients with low pressure glaucoma [19,20]. Pseudoexfoliation and steroid induced glaucoma may be found more commonly in this population because of a relatively more severe course either by damage or IOP, moving these forms of secondary open angle glaucoma into the higher GI groups. Patients with pseudoexfoliation typically have a larger IOP reduction after trabectome surgery [2] which may have contributed to the observed group behavior of a better pressure decrease in those groups in our study. GI4 had a significantly worse visual acuity than other groups which may be a result of commonly coinciding cataracts [21] that are still present in some of these patients but also with loss of visual acuity from glaucoma damage in advanced visual field loss. The later appears to be reflected in the significantly larger C/D ratio in GI4, the results of more advanced structural damage. Both regression analyses are reflective of the demographic and biometric observations and also point to Hispanic ethnicity as a group that is at an increased risk [22].

Limitations of this study are the use of categorical rather than continuous data for baseline IOP and a simplified stratification of glaucoma severity. We did this to avoid progressive underrating of glaucoma severity in the low pressure glaucoma range when raw data was used. The relatively large number of patients analyzed here and equal group sizes allowed to apply meaningful and valid statistical methods. Another shortcoming is that this study is retrospective in nature and may include bias for procedure selection. This is partially countered by relatively even patient numbers in all groups.

A recent study indicated that there is a tipping point during the course of glaucoma suggesting that more severe glaucoma may require more aggressive pressure lowering [23]. In this context, while this study can guide glaucoma surgeons to avoid risks inherent in traditional glaucoma surgeries, it is important to keep in mind that lowering intraocular pressure is a lifelong commitment to manage an often relentless and progressive disease. A minimally invasive surgery may only be the initial step.

## Supporting Information

S1 Fig IOP reduction at 1 year as percentage of preoperative IOP. A) In all open angle glaucoma patients, a higher glaucoma index group assignment indicating more severe glaucoma was found to be associated with a larger IOP reduction (percentage +/- 95% confidence interval). B) Primary open angle glaucoma had highly similar IOP percentage reduction at 1 year (percentage +/- 95% confidence interval).
(PDF)
