# Supplementary material for: Impact of a Glaucoma Severity Index on Results of Trabectome Surgery: Larger Pressure Reduction in More Severe Glaucoma
Source: PLoS One. 2016 Mar 23;11(3):e0151926. doi: 10.1371/journal.pone.0151926 (PMC4805208; doi:10.1371/journal.pone.0151926)
Supplement: S1 Fig — A) In all open angle glaucoma patients, a higher glaucoma index group assignment indicating more severe glaucoma was found to be associated with a larger IOP reduction (percentage +/- 95% confidence interval). B) Primary open angle glaucoma had highly similar IOP percentage reduction at 1 year (percentage +/- 95% confidence interval). (PDF) [file pone.0151926.s001.pdf]

S1 Fig.

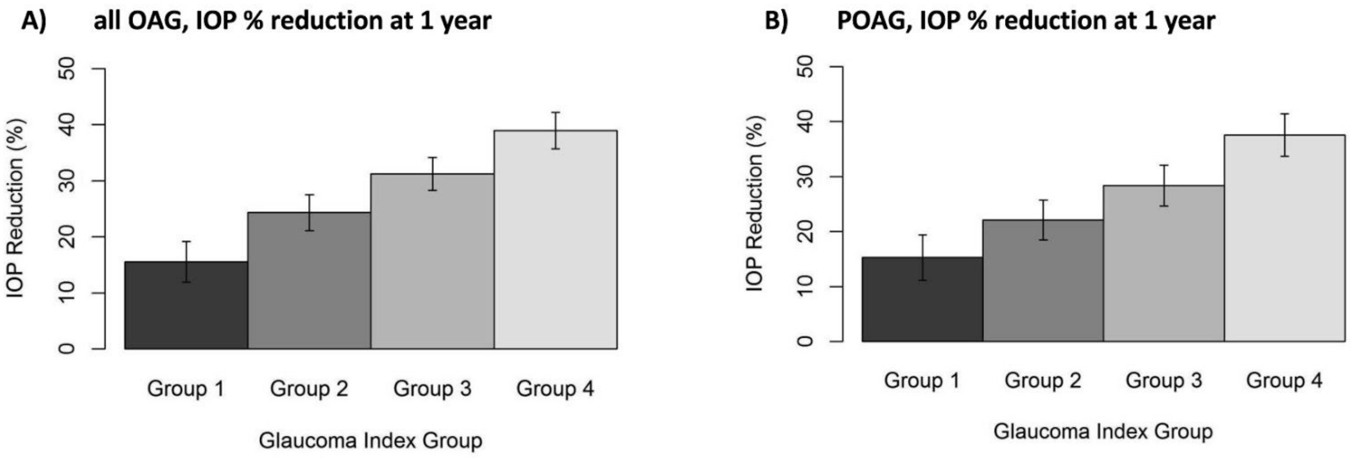

S1 Fig. IOP reduction at 1 year as percentage of preoperative IOP. A) In all open angle glaucoma patients, a higher glaucoma index group assignment indicating more severe glaucoma was found to be associated with a larger IOP reduction (percentage +/- 95% confidence interval). B) Primary open angle glaucoma had highly similar IOP percentage reduction at 1 year (percentage +/- 95% confidence interval).
